# Therapeutic success in a case of anaerobic sepsis with multiple brain abscesses in the context of an efficient interdisciplinary cooperation

**DOI:** 10.1186/1471-2334-13-S1-P36

**Published:** 2013-12-16

**Authors:** Anca Mihaela Drăgoi, Nicoleta Irimescu, Sorinela Ileana Diaconu, Felicia Georgescu, Doina Clementina Antonică, Radu Daniel Perin, Ana-Maria Petrescu

**Affiliations:** 1 National Institute for Infectious Diseases “Prof. Dr. Matei Balş”, Bucharest, Romania; 2 Carol Davila University of Medicine and Pharmacy, Bucharest, Romania; 3 Neurosurgery III Department, Clinical Emergency Hospital “Bagdasar Arseni”, Bucharest, Romania

## Background

Brain abscess is one of the most serious complications of head infections, with significant case-fatality rates. Great progress was made after introduction of antimicrobial therapy with improved microbiologic culture techniques, particularly for anaerobes, and stereotactic brain biopsy and aspiration techniques. The incidence of neurological sequelae in patients who survive a brain abscess is high.

## Case report

We present the case of a 42 year-old male patient with no significant past medical history, except for a wasting status caused by a neurotic depression; he was non-smoker, not addicted to alcohol, no HIV infection.

High fever, altered clinical state and mental status, right hemiplegia developed 4-5 days prior to admission to the Adults IV Department of the National Institute for Infectious Diseases “Prof. Dr. Matei Balş”, Bucharest.

Initial investigations diagnosed severe sepsis with multisystem organ failure, with positive blood cultures with *Fusobacterium nucleatum*, and multiple brain abscesses; simultaneously, a left renal abscess and a left paracardiac pneumonia were detected. Initial antibiotic (meropenem with vancomycin), anti-inflammatory (corticosteroids) and supportive therapy determined a good clinical and paraclinical evolution.

After 9 days a stereotactic CT-guided aspiration of pus from the brain abscess was performed and PLEX-ID screening identified *Fusobacterium necrophorum*.

Unfavorable neurological evolution imposed another brain abscess stereotactic CT–guided aspiration, with negative pus culture. After 28 days of treatment with the mentioned antibiotics switch therapy with metronidazole was performed. The complete duration of antibiotic therapy of 100 days in hospital period, and 20 days at home, led to diminishing the brain abscesses and the complete remission of the renal abscess. Although another brain abscess puncture was needed, the patient refused, and after discharge from the hospital he was lost to follow-up, returning after 8 months from the beginning of the disease. This time, clinical evolution and MRI showed a favorable evolution of the brain abscesses, except for one abscess, which needed another stereotactic CT–guided aspiration, with good results. Neurosurgery consultation concluded, in context of normal paraclinical investigations, that there was no need for antibiotic therapy.

## Conclusion

The prognosis of patients with bacterial brain abscess is determined by the rapidity of progression of the disease before hospitalization and the patient’s mental status on admission. In our case, with double anaerobic bacteria etiology and multiple determinations, interdisciplinary collaboration was essential for a good outcome.

